# De-Novo Assembly and Analysis of the Heterozygous Triploid Genome of the Wine Spoilage Yeast *Dekkera bruxellensis* AWRI1499

**DOI:** 10.1371/journal.pone.0033840

**Published:** 2012-03-28

**Authors:** Chris D. Curtin, Anthony R. Borneman, Paul J. Chambers, Isak S. Pretorius

**Affiliations:** 1 The Australian Wine Research Institute, Adelaide, Australia; 2 The University of South Australia, Adelaide, Australia; Institut de Genetique et Microbiologie, France

## Abstract

Despite its industrial importance, the yeast species *Dekkera (Brettanomyces) bruxellensis* has remained poorly understood at the genetic level. In this study we describe whole genome sequencing and analysis for a prevalent wine spoilage strain, AWRI1499. The 12.7 Mb assembly, consisting of 324 contigs in 99 scaffolds (super-contigs) at 26-fold coverage, exhibits a relatively high density of single nucleotide polymorphisms (SNPs). Haplotype sampling for 1.2% of open reading frames suggested that the *D. bruxellensis* AWRI1499 genome is comprised of a moderately heterozygous diploid genome, in combination with a divergent haploid genome. Gene content analysis revealed enrichment in membrane proteins, particularly transporters, along with oxidoreductase enzymes. Availability of this assembly and annotation provides a resource for further investigation of genomic organization in this species, and functional characterization of genes that may confer important phenotypic traits.

## Introduction

The yeast species *Dekkera (Brettanomyces) bruxellensis* is predominantly associated with wine, beer, and cider, but has also been detected in kombucha (sweet tea) and sourdough fermentations [1], [2]. In light of this association with fermentation environments, it is unsurprising that recent studies have highlighted the potential for this yeast species to be harnessed in industrial bio-ethanol production processes [3]–[5].

The nearest phylogenetic neighbour of *D. bruxellensis* is *Dekkera anomala*, a species that has been found to co-exist with D. bruxellensis in some niches, such as cider fermentation [6], but has never been unambiguously isolated from wine [7]. *D. bruxellensis is* well adapted to this environment. Some of these adaptations; osmotolerance, ethanol tolerance, acid tolerance, are shared with *Saccharomyces cerevisiae* - the ubiquitous fermentation yeast that precedes *D. bruxellensis* in population ecology of the industrial fermentation systems mentioned above. In addition, *D. bruxellensis* is both petite and crabtree positive, and has convergently evolved the make-accumulate-consume lifestyle in parallel with *S. cerevisiae* [8]. However, *D. bruxellensis* is capable of surviving and proliferating for extended periods of time after *S. cerevisiae* has completed alcoholic fermentation and its population declined [9].

It is well accepted that D. bruxellensis is the key wine microorganism that converts hydroxycinnamic acids present in grape must and wine into the volatile phenols 4-ethylphenol (4-EP), 4-ethylguaiacol (4-EG) and 4-ethylcatechol (4-EC). Wine containing above-threshold concentrations of these compounds exhibits ‘Brett’ character [10], which can result in wines that are less-preferred by consumers [11]. Spoilage of wine by D. bruxellensis is, in fact, considered the foremost microbiological issue in the wine industry [12]. In some industries the presence of D. bruxellensis is desirable. For example, the Belgian lambic and gueze ales are typified by flavours generated during secondary fermentation by this yeast [13] through production of the same volatile compounds (4-EP and 4-EG). This dichotomy is in part due to the difference in relative concentration of these volatile phenols - beer generally contains higher concentrations of 4-EG (clove-like, or spicy aroma), while wine contains more 4-EP (medicinal aroma).

Partial survey sequencing of the *D. bruxellensis* CBS 2499 genome [14] revealed an enrichment of genes encoding transporters and enzymes associated with nitrogen and lipid metabolism, which may go some way to explaining the capacity of this yeast to survive in the relatively barren post-alcoholic fermentation environment. However, due to the incomplete nature of these data, the genomic organization for this species is less certain. Re-analysis revealed the previously assumed haploid status for CBS 2499 was incorrect, and instead it appeared to be a relatively heterozygous diploid/polyploid [15]. Extensive karyotype variability was also observed among *D. bruxellensis* strains in the same study, leading the authors to question existence of a sexual cycle for this species.

Despite its importance in food and beverage industries, very little is known regarding the genetics of this microorganism. Next-generation genomic sequencing is providing the means of fast tracking genetic analysis of intractable, non-model organisms, by enabling direct access to genetic make-up, and subsequent functional analysis. To gain insight into the genome features and gene content of *D. bruxellensis*, we have sequenced the prevalent wine spoilage strain, AWRI1499 [7]. We found *D. bruxellensis* AWRI1499 has a triploid genome, that relative to phylogenetically related species is enriched in genes that may aid survival in the challenging environment of wine.

## Results and Discussion

### Genomic assembly and annotation

De-novo auto-assembly of sequence reads yielded 26,043 contigs, with N50 of 7 kb at 9.7-fold coverage, and an assembly length of 40.2 Mb. The genome size of *D. bruxellensis* was estimated at between 20 and 30 Mb by pulsed-field electrophoresis of several European strains [14]. This estimation, in combination with paired-end information present in our assembly suggested that many small contigs had not been incorporated into larger contigs by the assembler due to heterozygosity. Manual editing of the assembly was therefore used to reassemble contigs into larger scaffolds under the assumption that AWRI1499 was diploid. Manual finishing yielded a 12.7 Mb assembly comprised of 324 contigs (N50 = 68 kb) in 99 scaffolds, at median coverage of 26-fold (see Table S1 for contig statistics). ‘Unlocated’ contigs remaining after this process that were >500 bp in length, and consisted of at least 10 sequence reads, were found to harbour at least 22 Ty3-gypsy-like, and 43 Ty1-copia-like transposable elements. AT-rich contigs AWRI1499with high read depth were designated as putative mitochondrial genome sequences and separated from the genomic assembly. Assembly of these contigs against the mitochondrial genome recently published for *D. bruxellensis* CBS 2499 [16] resulted in almost complete coverage (99.7%) and high levels of nucleotide identity (>98% across 20 kb alignments) (data not shown). Average coverage for these contigs was 225-fold, suggesting approximately nine copies of the mitochondrial genome per *D. bruxellensis* cell.

RNA samples pooled from cultures grown under standard laboratory conditions, and in the presence of 2 mM ferulic acid [17], were sequenced and mapped to the genomic assembly to provide information on gene positions. Gene model prediction was conducted using Augustus [18], taking exon hints based upon this mapped RNAseq data, and predicted proteins annotated using Blast2go [19]. Of the 4969 gene models predicted by Augustus, 688 were not assigned a significant match by Blast2go against the NCBI non-redundant protein database. Median length of un-annotated gene models was 531 nucleotides, compared with 957 nucleotides for annotated gene models. When translated, 14.5% of gene models lacking annotation yielded proteins shorter than 100 amino acids, and may therefore be considered spurious. Consequently, there are 588 gene models in this assembly coding for orphan proteins, making up 11.8% of *D. bruxellensis* AWRI1499 predicted proteins.

To test the AWRI1499 genome assembly for completeness, the core eukaryote gene (CEG) set of 248 proteins from six reference species [20] were used as translated nucleotide blast (tblastn) queries against the AWRI1499 predicted open reading frames (ORFs). 213 CEG proteins were detected (E-values<1e-20), while a further 20 CEGs were found by tblastn against the AWRI1499 scaffold assembly, suggesting the Augustus gene model prediction failed to detect around 10% of potential ORFs. Four CEGs (corresponding to *S. cerevisae* ORFs YGR181w, YOR103c, YGL231c, YGL047w) were detected at low identity or had only partial matches in the scaffold consensus. A further 11 CEGs with AWRI1499 gene model tblastn hits at worse than >1e-20 were confirmed by tblastn of non-*S.cerevisiae* fungal orthologs of each protein against the scaffold consensus. This analysis suggested the genomic assembly of AWRI1499 was approximately 98% complete.

### Phylogenetic position of D. bruxellensis AWRI1499

**Figure 1:**
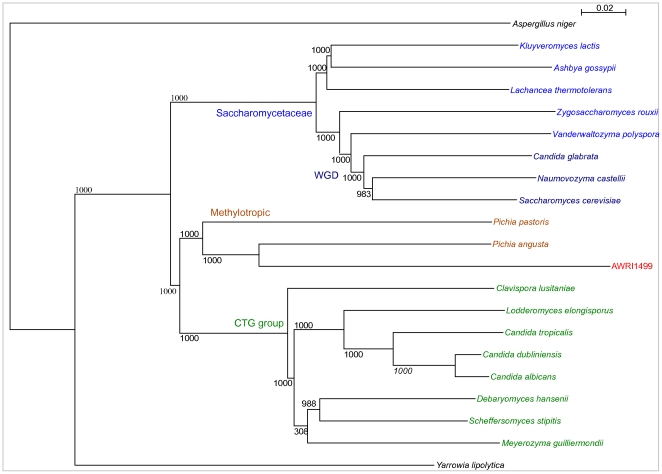
Phylogeny for species most represented in blastp searches for *D. bruxellensis* AWRI1499 proteins. A Maximum likelihood tree was generated from an amino-acid alignment of 542 putative orthologous proteins. Bootstrap values (1000 randomisations) for all nodes shown.

The 20 fungal species most represented as blastp top-hits for AWRI1499 gene models (Figure S1) were selected for phylogenetic analysis. Protein sequences were obtained for each species and pair wise protein blasts performed. A total of 542 putative orthologous proteins present in all species (as defined by E-values of less than 1 e-20) were aligned separately and then concatenated into a single hybrid sequence, from which a maximum-likelihood phylogeny was constructed (Figure 1). The topology of this phylogeny reflected the top-hit statistics (Figure S1), with *Pichia angusta* the closest relative to *D. bruxellensis* among those fungi for which sequenced genomes are available. Along with *Pichia pastoris*, these species, while not closely related, form a grouping distinct from the whole genome duplication and CTG clades, respectively.

### SNP density and haplotype sampling

**Figure 2:**
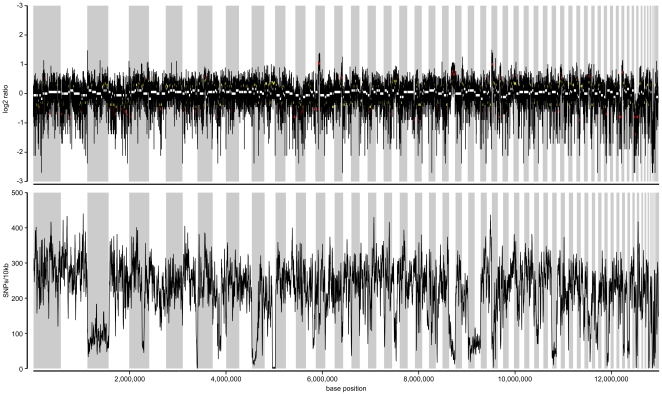
Sequencing coverage and SNP density of *D. bruxellensis* AWRI1499 genomic assembly. Coverage (log2 ratio of overall median) and SNP density (per 10,000 nucleotides) determined for each contig using a sliding window of 1001 bp, with a 100 bp step frequency, and plotted in scaffold order. Scaffold boundaries are indicated by alternate shading, regions of copy number variation greater than 1.25- or 1.5fold indicated by yellow, and red dots, respectively. White dots indicate coverage consistent with overall genomic median.

Significant heterozygosity was observed in the assembly, with a median of 27 single nucleotide polymorphisms (SNPs) per 1000 nucleotides - a total of 342,900 sites across the genome. However, this density was not uniform across the assembly with some regions displaying substantially reduced rates of heterozygosity (Figure 2). In particular, contigs comprising scaffold 3 of the assembly displayed a significantly reduced SNP density.

**Figure 3:**
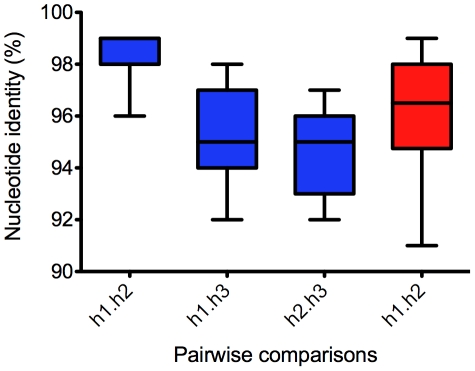
Pairwise comparison of ORF haploptypes. Manually extracted haplotypes were aligned, and pairwise nucleotide identity calculated. For ORFs represented by three haplotypes, the pair with highest nucleotide identity were assigned to haplotype 1 (h1) and haplotype 2 (h2).

Within predicted open reading frames there were fewer SNPs than seen for the entire genome, with an average of 1.9%. The majority of these coding region SNPs were synonymous Nonetheless 0.66% of all nucleotides are predicted to produce non-synonymous amino acid substitutions across *D. bruxellensis* ORFs. During assembly it was noted that in many regions more than two distinct sequences were evident amongst sequence reads. To investigate the distribution of coding region SNPs across multiple alleles, a subset of ORFs (60 of 4969) with very high SNP density (6%), high SNP density (4%), and moderate SNP density (2%) were randomly sampled across the genome, and manually separated into haplotypes. Of the 60 ORFs sampled, 37 could be resolved into three distinct haplotypes. ORFs from which two haplotypes were resolved did not originate only from regions with low SNP density, such as scaffold 3, but were dispersed across the assembly (data not shown). The probability of resolving three haplotypes from ORFs with 2% SNP density was, however, significantly lower (P = 0.0147, Pearson Independence Test). Pair wise nucleotide sequence alignments (Figure 3) revealed that for most loci two haplotypes were closely related (∼99% identity), while the third displayed significantly greater sequence divergence (∼95% identity). Nucleotide identity at loci where only two haplotypes were recovered was found to span a range from 91–99%, indicating that a similar level of overall sequence divergence was captured in these two haplotypes as was found in those that could be separated into three distinct alleles. This genome assembly does not permit haplotype phasing, however the most parsimonious explanation for the presence of three alleles for these genes (two closely related and a third more diverged) would be that the genome of *D. bruxellensis* AWRI1499 comprises a moderately heterozygous diploid genome, in addition to a divergent haploid genome. In combination with observations for a very limited number of genes from other strains of *D. bruxellensis* [15], this data indicates that *D. bruxellensis* arose through the hybridisation of two closely related species (one diploid, one haploid). Regions of reduced SNP density, such as scaffold 3, would therefore most likely reflect a partial replacement of the divergent haploid by the diploid genome, a phenomenon observed in *Saccharomyces* interspecific hybrids [21]. Furthermore, mechanisms that drive genome stabilisation in such hybrids [22] could explain the extreme karyotypic variability observed for *D. bruxellensis* [15]. Whilst the triploid status of *D. bruxellensis* does not by default infer sterility [23], along with the observation of extreme karyotypic variability displayed by this species [15], it is likely that *D. bruxellensis* lacks a fully functional sexual cycle.

### Gene content analysis

**Figure 4:**
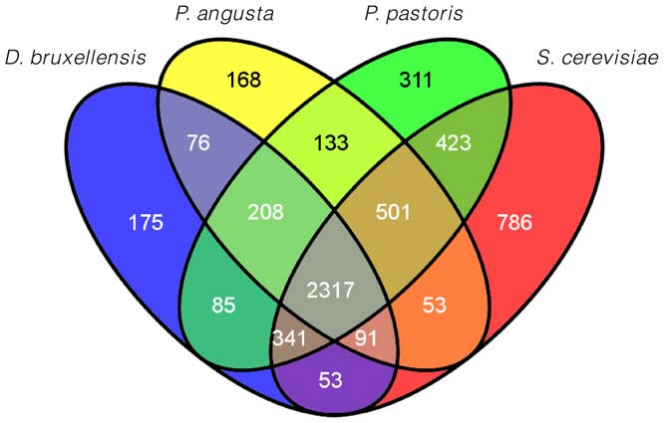
Venn diagram of OrthoMCL cluster distribution across *D. bruxellensis*, *P. angusta*, *P. pastoris*, and *S. cerevisiae*.

**Figure 5:**
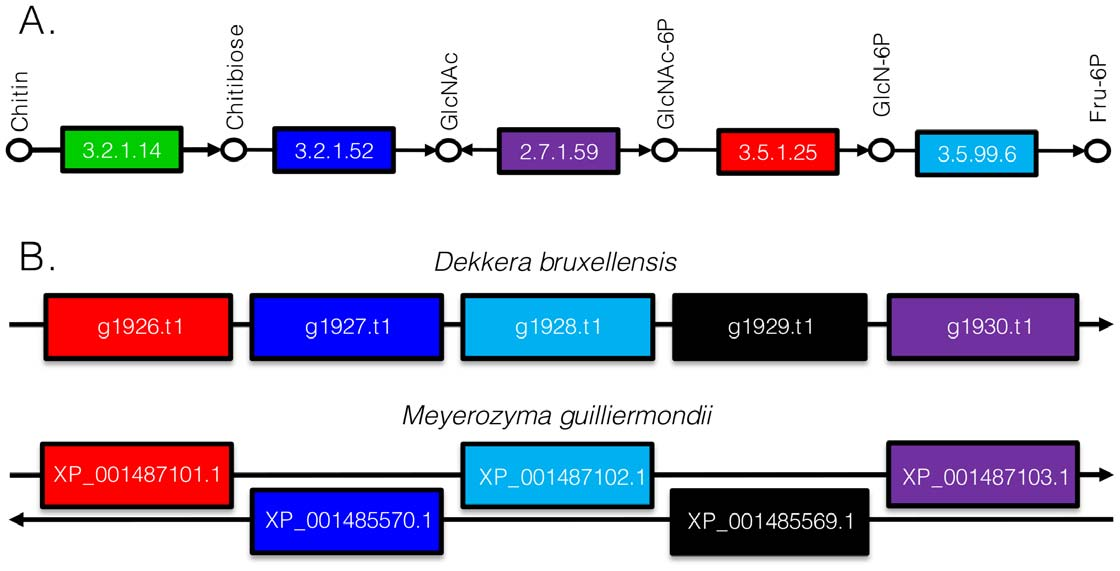
Metabolic capability of *D. bruxellensis* revealed through OrthoMCL analysis. Chitin/N-acetylglucosamine assimilation pathway (A) and arrangement of gene cluster in D. bruxellensis AWRI1499 and *M. guilliermondii* (B). Pathway redrawn from KEGG map (ko00520). ORF numbers for *D. bruxellensis* proteins and accession numbers for *M. guilliermondii* orthologs mapped to chitin/N-acetylglucosamine assimilation pathway, indicated by shared box colour. Chitinase (3.2.1.14) not located in cluster, g1929.t1 & XP_001485569 members of MFS transporter superfamily.

To ascertain if there were any genes which could provide insight into the evolutionary history of *D. bruxellensis*, the predicted proteome of AWRI1499 was submitted to OrthoMCL for assignment to orthologous clusters (Table S2), along with the proteomes of *P. angusta*, *P. pastoris*, and *S. cerevisiae*. While the two *Pichia* strains were included for analysis due to their evolutionary relationship to *D. bruxellensis*, *S.* cerevisiae, which is distantly related to *D. bruxellensis*, was included as these two species occupy very similar environmental niches (alcoholic fermentation) and are hypothesised to have undergone convergent evolution of several phenotypic traits [8]. Figure 4 summarises the number of OrthoMCL clusters shared between species. Within the 175 clusters unique to *D. bruxellensis* were 198 predicted proteins. Seventy-seven of these proteins were not annotated by Blast2go which has a more stringent blastp cut-off (E<1e-6). A further 64 were found to have a blastp match to a protein from one of the other three species (assigned to a different OrthoMCL cluster) and there were 10 dubious assignments of short predicted proteins (<100 amino acids). Excluding four transposon-like proteins, there were no significantly enriched GO-terms for the remaining 43 proteins (Fisher's exact test, FDR 0.05). Nonetheless, some of these may contribute to the ‘scavenging’ lifestyle of this yeast. Several proteins involved in carbon source utilisation (chitin, *N*-acetylglucosamine, galactose, mannose, lactose), were detected. Interestingly, most genes required for chitin/*N*-acetylglucosamine catabolism were found to be present in a single cluster of five genes, which were also found to be present in *M. guilliermondii*, although separated in two blocks of synteny (Figure 5). This pathway may represent efficient recycling of cell wall building blocks when required, or provide access for *D. bruxellensis* to an abundant carbon source in fungal communities.

To detect instances of conserved gene family expansion during the evolution of *D. bruxellensis*, a total of 2,317 OrthoMCL clusters that were represented by at least one protein in all four species were examined for either: expansion specifically in *D. bruxellensis*, lineage-specific expansion (expansion relative to *S. cerevisiae* but not *P. angusta* or *P. pastoris*) and expansion driven by convergent evolution (expansion relative to *P. angusta* and *P. pastoris*, but not *S. cerevisiae*). *D. bruxellensis* proteins corresponding to expanded clusters were then examined for GO-term enrichment using Blast2go, with significantly over-represented terms (Fisher's exact test, FDR<0.05) summarised in Figures S2, S3, S4, S5, S6, S7, S8, S9 and S10. This analysis also revealed significantly under-represented terms, highlighting which process and functions were less likely to contribute specific phenotypic traits of *D. bruxellensis*.

Proteins corresponding to OrthoMCL clusters expanded in *D. bruxellensis* were highly enriched in GO terms corresponding to the cell membrane. OrthoMCL cluster OG5_126579 was quantitatively the most expanded in *D. bruxellensis*. This cluster, to which *S. cerevisiae* ORFs *FIG2, FLO1, FLO5, FLO9, HKR1, HPF1, MSB2, MUC1, PRM7, YIL169C*, and *YMR317W*, were assigned, encompasses divergent plasma membrane and cell wall proteins involved in cell wall budding, adhesion, and pseudohyphal growth. *D. bruxellensis* has been shown to form biofilms [24] and pseudohypae [25] - traits that would be advantageous for survival in wine stored in oak barrels [10] where they would provide the means of adhering to the internal wall of the barrel and resistance to removal by high pressure cleaning. Membrane associated transporters were also significantly expanded in *D. bruxellensis*. Amino acid permeases were expanded compared to all three species, while the *D. bruxellensis/Pichia* lineage expansion was enriched in transmembrane drug transporters, and *D. bruxellensis/S. cerevisiae* expanded clusters were enriched in nucleobase transporters. Survey sequencing of *D. bruxellensis* strain CBS 2499 also revealed an enrichment of transporters, consistent with adaptation to relatively low nutrient environments like wine [14].

Despite exposure to the common wine preservative, sulfite, *D. bruxellensis* AWRI1499 has only one ORF (g80.t1) that encodes a protein with homology to the *S. cerevisiae* sulfite efflux transporter, Ssu1p. This protein confers sulfite tolerance [26] and has been the subject of positive selection in vineyard *S. cerevisiae* populations exposed to sulfite [27]. In *S. cerevisiae*, *SSU1* is duplicated, and strains with high levels of sulfite tolerance harbor one or more *SSU1-R* (resistant) alleles [28] that are highly expressed. Furthermore, a sulfite inducible *SSU1-R* allele was recently described for the wine yeast *S. cerevisiae* 71B [29]. Genomic and transcriptomic comparison of multiple *D. bruxellensis* strains will be required to determine whether molecular mechanisms conferring sulfite tolerance are similar for the two species.

**Figure 6:**
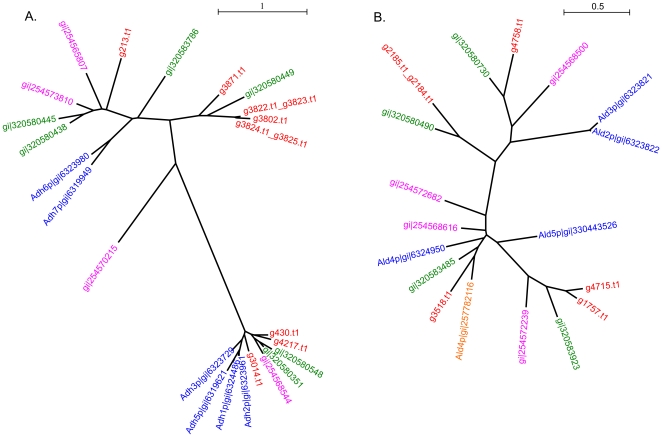
OrthoMCL oxidoreductase clusters expanded in *D. bruxellensis*. Alcohol dehydrogenase (A). and aldehyde dehydrogenase (B). Unrooted maximum likelihood trees generated from amino acid alignments of proteins assigned to the same OrthoMCL cluster. Red = *D. bruxellensis*, blue = *S. cerevisiae*, green = *P. angusta*, pink = *P. pastoris*, orange = *Issatchenkia terricola*.

GO terms corresponding to oxidoreductase catalytic activity were also a feature in OrthoMCL clusters expanded in all comparisons. This may reflect a strategy evolved to enable survival in anaerobic conditions where the species has impaired capacity to regenerate NAD(P)+. *D. bruxellensis* contains eight oxidoreductases corresponding to the OrthoMCL cluster of alcohol dehydrogenase proteins (Figure 6A), and five corresponding to aldehyde dehydrogenase (Figure 6B), while also showing expansion in other, less specific, oxidoreductase clusters. Oxidation of acetaldehyde to acetate, and possible imbalance in subsequent acetyl-CoA and/or succinate formation, has been linked to overproduction of acetate under aerobic conditions, and the Custers effect (inhibition of fermentation under anaerobic conditions) [30]. In this context it is interesting to note that the closest protein sequence match for the *D. bruxellensis* Ald4p ortholog is an acetaldehyde dehydrogenase recently cloned and characterised from *Issatchenkia terricola*, which was found to be significantly more active than *S. cerevisiae* aldehyde dehydrogenase enzymes in metabolising acetaldehyde [31].

**Figure 7:**
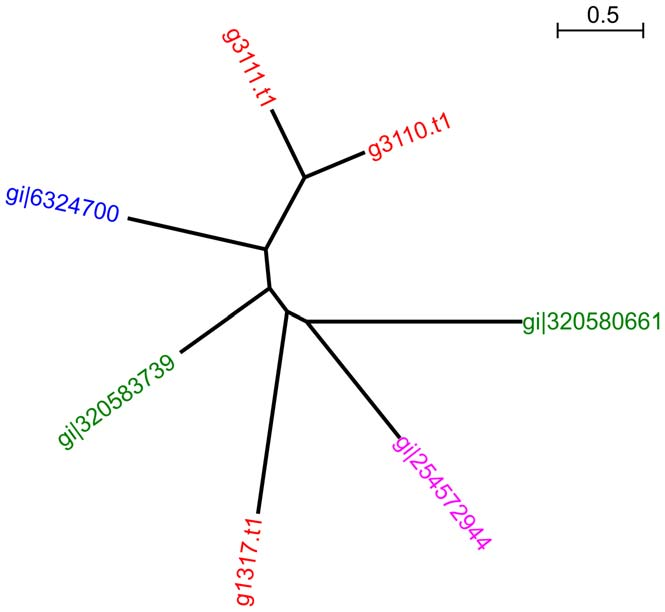
OrthoMCL cluster expanded in *D. bruxellensis*, corresponding to isoamyl-acetate hydrolase. Unrooted maximum likelihood trees generated from amino acid alignments of proteins from each species assigned to the same OrthoMCL cluster. Red = *D. bruxellensis*, blue = *S. cerevisiae*, green = *P. angusta*, pink = *P. pastoris*.

In *S. cerevisiae*, alcohol and aldehyde dehydrogenases, along with other oxidoreductase enzymes, contribute to the formation of aroma compounds through the Ehrlich pathway [32]. The balance between oxidation and reduction determines, for example, formation of isoamyl-alcohol and its well known ester isoamyl-acetate, or alternatively isovaleric acid. *D. bruxellensis* has been reported to produce the latter compound, which has a ‘rancid’ aroma thought to contribute to the sensory impact of *D. bruxellensis* on wine [33]. In this context it is worth noting that while hydrolase OrthoMCL clusters were not significantly expanded according to the GO-term enrichment analysis, three *D. bruxellensis* proteins were found that each have homology to isoamyl-acetate hydrolysis enzymes (Figure 7). Two homologs present as a tandem repeat (g3110.t1, g3111.t1) were most similar to the *S. cerevisiae* Iah1p enzyme which, in conjunction with Atf1p, determines concentrations of isoamyl alcohol and isoamyl acetate that are produced during fermentation [34].

A key trait of *D.bruxellensis* is the capacity to take up hydroxycinnamic acids and convert them to their respective ethylphenols [10]. This metabolic pathway requires an enzyme with phenolic acid decarboxylase (PAD) activity, an activity found across many fungal and bacterial species, and an enzyme with vinylphenol reductase (VPR) activity. Aside from *D. bruxellensis*, the only other wine-associated yeast species demonstrated to convert vinylphenols to ethylphenols through this enzymatic activity is *M. guilliermondii* [35].

Tchobanov et al. [36] partially purified a 26 kDa protein that exhibited VPR activity, and assembled a putative VPR ORF from CBS 2499 [14] contigs against peptide fragment sequences. Blastn of DbVPR against the AWRI1499 ORFs revealed a close match (99% nucleotide identity) with g4418.t1. This protein was, however, annotated by Blast2go as a carboxypeptidase-y inhibitor, of the phosphatidylethanolamine-binding protein (PEBP) family, sharing 58% amino acid identity with orthologous protein from *P. angusta*. Proteins of this family do not have NAD(H) or NADP(H) binding domains, thus it seems questionable that DbVPR indeed performs the VPR function. Godoy et al. [37] also reported purification of a putative VPR enzyme from *D. bruxellensis*, which exhibited very different kinetic properties. Finding the gene that encodes this critical enzyme in *D. bruxellensis* may be possible by functional analysis of the oxidoreductase OrthoMLC clusters expanded in *D. bruxellensis*.

**Figure 8:**
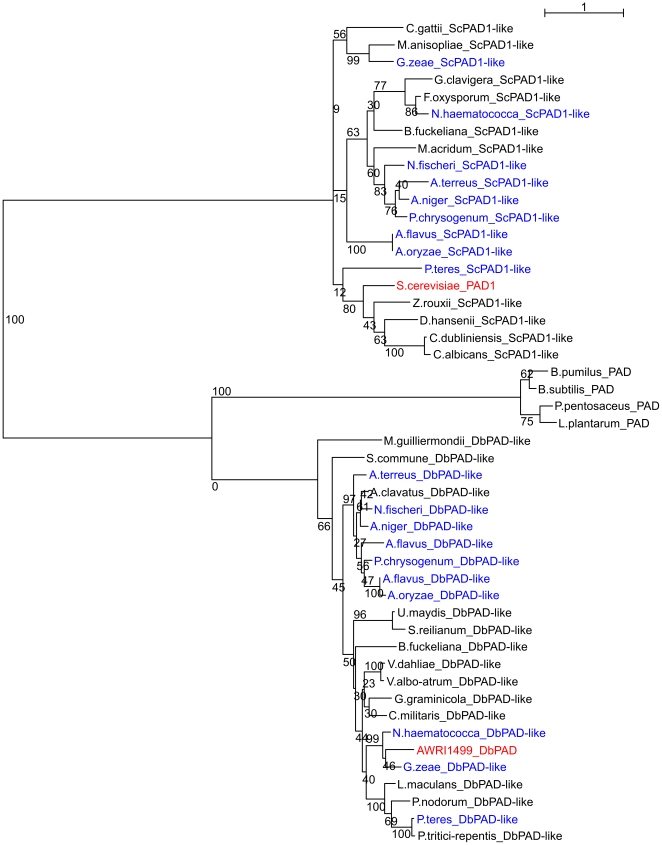
Phylogeny of fungal phenolic acid decarboxylase enzymes. Maximum likelihood tree generated from amino acid alignment of all fungal proteins with homology to *S. cerevisiae* Pad1p (ScPAD1) or putative *D. bruxellensis* Padp (DbPAD). Species with homologs of both proteins indicated in blue. Bootstrap values (100 randomisations) for all nodes shown.

Characterised proteins with PAD activity belong to two InterProScan families; phenylacrylic acid decarboxylase (IPR004507) which includes *S. cerevisiae* Pad1p, and the bacterial phenolic acid decarboxylases (IPR008729). No homolog of the *S.cerevisiae* protein Pad1p was found in the partial sequence of *D. bruxellensis* CBS 2499 [14], nor did we find one in the genomic assembly of AWRI1499. We therefore screened the *D. bruxellensis* genome sequence for potential PAD-encoding genes using characterised bacterial proteins from *Bacillus pumulis, Bacillus subtilis, Pediococcus pentosaceus* and *Lactobacillus plantarum*. No sigificant hits were found amongst the *D. bruxellensis* ORFs, however a significant tblastn hit (E<1e-30) was found in contig 3340 of scaffold 100. A hypothetical protein, DbPad, was generated by fusing two frameshifted (due to assembly sequence errors) translations, and aligned against fungal proteins with homology to DbPad or *S. cerevisiae* Pad1p. The resultant maximum likelihood tree (Figure 8) shows that several ascomycete and basidomycete proteins share homology with the putative DbPad, which is more similar to bacterial phenolic acid decarboxylase proteins than the *S. cerevisiae* Pad1 protein. The tree also highlights that several filamentous fungi have both Pad1p- and DbPad-like proteins.

PAD activity in *D. anomala* was attributed to a protein with homology *to S. cerevisiae* Pst2p, a member of the flavodoxin family [38]. Three *D. bruxellensis* AWRI1499 ORFs encode Pst2 homologs, exhibiting 99% (g3584.t1), 67% (g3585.t1) and 57% (g797.t1) amino acid similarity to the *D. anomala* Pst2 protein. These proteins do not share homology with the phenylacrylic acid decarboxylase, or phenolic acid decarboxylase families. Nonetheless, it is possible that through duplication of Pst2 in *D. bruxellensis* AWRI1499 and subsequent functional divergence, PAD activity can be attributed to one of the three proteins. On the other hand, it is interesting to note that *M. guilliermondii* was the only budding yeast found to contain a DbPad homolog (Figure 8). An enzyme with phenolic acid decarboxylase activity was recently purified from *M. guilliermondi* [39], and found to have a molecular mass of 20 kDa, the predicted size of the DbPad protein. Furthermore, this protein exhibited a substrate specificity towards hydroxycinnamic acids favouring *p*-coumaric acid and ferulic acid over caffeic acid. Caffeic acid is typically more abundant than *p*-coumaric acid in wine, particularly when esterified with tartaric acid [40]–[42], whereas wine affected by *D. bruxellensis* exhibits higher concentrations of 4-ethylphenol (derived from *p*-coumaric acid) than 4-ethylcatechol [43]. We can, therefore, speculate that the dominant sensory characteristics attributed to *D. bruxellensis* spoilage of wine are a consequence of DbPad substrate specificity. Availability of the AWRI1499 genomic assembly and annotation will facilitate functional characterisation of the DbPAD gene, and others that may confer industrially relevant phenotypic traits.

### Conclusion

This study sheds light on an industrially important microorganism which, because of its intractable nature, has been poorly understood. Analysis of gene content points to an organism that is metabolically well equipped to survive in challenging environments such as wine. The genomic assembly described in the current study reveals a triploid genome, consistent with speciation through inter-specific hybridisation and an asexual lifestyle. Availability of the genomic assembly and annotation will facilitate advancement in our knowledge of *D. bruxellensis* biology, both from an evolutionary perspective and in terms of its role in fermentation processes.

## Materials and Methods

### Yeast strain, nucleic acid preparation, and sequencing

*D. bruxellensis* strain AWRI1499 was obtained from The Australian Wine Research Institute Microorganisms Culture Collection. Genomic DNA was prepared using a standard zymolyase and phenol-chloroform extraction from cultures grown under standard conditions. RNA was extracted from cultures grown under standard conditions and incubated for 2 h in the presence of 2 mM ferulic acid to induce a stress response [44], using Trizol (Invitrogen), RNase-free DNAse (Qiagen) treatment, and purified using the Purelink RNA Mini Kit (Invitrogen). Shotgun and paired-end library construction and sequencing of genomic DNA was performed by 454 Life Sciences, A Roche Company (Branford, CT) using GS-FLX Titanium series chemistry and their own standard protocols. Of 1,859,751 total reads generated, 1,227,460 were paired-end. Average read lengths were 345 bp for the paired-end library and 369 bp for the shotgun library. RNAseq was performed at The Ramaciotti Centre for Gene Function Analysis (Sydney, NSW) using 2×100 bp paired end libraries on the Illumina HiSeq2000.

### De-novo genome and transcriptome assembly

MIRA (v3rc2) [45] was used to assemble shotgun and paired-end reads. The resultant assembly file, comprising 84% of sequencing reads, was imported into Lasergene Seqman (v8) for manual finishing. All vs all Blastn was performed for ‘small’ contigs and ‘large’ contigs (arbitrary size cutoff), and a custom python script developed that sequentially unlocked reciprocal best-hit contigs (E-value<1e-30) in Seqman, and reassembled the ‘small’ contig into the matching ‘large’ contig, before locking all contigs. ‘Small’ contigs remaining after this process were reverse-complemented and reassembled against their reciprocal blastn best-hit large contig. Through this process the number of contigs was reduced to ∼6,000. Default Seqman parameters were used during manual assembly (match size 12, min match % 80, min seq length 100, max added gaps per kb 70, gap penalty 0.00, gap length penalty 0.70). Further manual assembly was guided by paired end information that inferred either an end-to-end join, extending length of contig/scaffold, or an internal assembly of a small contig into a larger contig. Blastn alignments were performed to determine appropriate course of action (align, assemble, or reverse-complement then assemble). Each reassembled contig was manually examined for consistency of paired-end data. Consensus sequences were exported with SNP detection at 50% of reads, and reimported into Seqman for subsequent alignment of INDEL-containing contigs. RNAseq reads were pooled and assembled using the Trinity pipeline [46], then transcript contig sequences imported into Seqman and assembled against the reference genomic assembly, to aid homopolymer detection and correction in putative open reading frames. This Whole Genome Shotgun project has been deposited at DDBJ/EMBL/Genbank under the accession AHIQ00000000. The version described in this paper is the first version, AHIQ01000000.

Regions of copy number variation were determined by calculating the per-base sequencing coverage across each sequencing contig with median smoothing (1001 bp window, 100 by step size). The ratio between coverage at each genomic location and overall median genomic coverage was calculated to determined the level of over- or under-representation for each location. Large scale aneuploidies were detected by screening for regions where median ratio for a contiguous stretch of at least 101 individual segments differed from the overall genomic median by either 1.25- or 1.5-fold.

### Open reading frame prediction and annotation

Open reading frame prediction was performed using Augustus v2.5.5 [18], incorporating open reading frame hints generated by mapping of RNAseq data to the MIRA assembly using BLAT. *S. cerevisiae* and *P. pastoris* gene models built in Augustus were compared for prediction of *D. bruxellensis* ORFs, with the *S. cerevisiae* based model resulting in a higher number of predicted ORFs and therefore preferred. ORFs were visualised in IGV v2.03 [47] with mapped RNAseq reads in order to identify and correct erroneous frameshifts due to 454 homopolymers (editing performed in Seqman). ORFs were then annotated using Blast2go v2.5 [19]. A custom perl script was used to evaluate codon usage and proportion of non-synonymous SNPs in ORFs.

### Phylogenetic analysis

Twenty species were identified in Blast2go as most frequent ‘top-hit’ in blastp searches conducted using the *D. bruxellensis* predicted proteins. The proteome for each species was used in pairwise blastp against *D. bruxellensis*, and reciprocal top-hits identified as putative orthologs. A table of putative orthologs across all 21 species is available in supplementary data (Table S3). Proteins present across all species (542) were separately aligned using the MUSCLE algorithm in Seaview [48], concatenated, then submitted to PhyML [49] for construction of a maximum likelihood tree, with 1000 randomisations. Phylogenetic analysis of specific proteins were also performed using this approach.

### Comparative ortholog cluster analysis

The predicted proteomes of *D. bruxellensis*, *P. pastoris, P. angusta*, and *S. cerevisiae* were submitted to the OrthoMCL web server [50] for assignment to orthologous clusters. Overlapping (and unique) clusters were identified using Venny [51] Lists of *D. bruxellensis* ORFs from OrthoMCL clusters found to be unique or expanded in the four-way comparision were manually curated for instances where sequential open reading frames in AWRI1499 were assigned to the same cluster. If these ORFs represented partial hits to different parts of the same protein in blast2go, or the sequential ORFs were within a region encompassed by tblastn of the relevant blast2go top-hit against the scaffold assembly, the sequential ORFs were assumed to comprise a single gene. Curated ORF lists were then tested for GO-term enrichment using Fisher's exact test in Blast2go.

## Supporting Information

Figure S1 **Distribution of Blastp top-hits against non-redundant database performed by Blast2go for all predicted** ***D. bruxellensis*** **AWRI1499 proteins.**
(TIF)

Figure S2 **GO-term enrichment analysis for OrthoMCL clusters common to** ***D. bruxellensis, P. pastoris, P. angusta,*** **and** ***S. cerevisiae*** **.** Biological process GO-terms enriched in comparison to all three *spp*.
(PDF)

Figure S3 **GO-term enrichment analysis for OrthoMCL clusters common to** ***D. bruxellensis, P. pastoris, P. angusta*** **, and** ***S. cerevisiae*** **.** Cell component GO-terms enriched in comparison to all three *spp*.
(PDF)

Figure S4 **GO-term enrichment analysis for OrthoMCL clusters common to** ***D. bruxellensis, P. pastoris, P. angusta*** **, and** ***S. cerevisiae*** **.** Molecular function GO-terms enriched in comparison to all three *spp*.
(PDF)

Figure S5 **GO-term enrichment analysis for OrthoMCL clusters common to** ***D. bruxellensis, P. pastoris, P. angusta*** **, and** ***S. cerevisiae*** **.** Biological process GO-terms enriched in comparison to *P. pastoris* and *P. angusta*, but not *S. cerevisiae*.
(PDF)

Figure S6 **GO-term enrichment analysis for OrthoMCL clusters common to** ***D. bruxellensis, P. pastoris, P. angusta*** **, and** ***S. cerevisiae*** **.** Cellular component GO-terms enriched in comparison to *P. pastoris* and *P. angusta*, but not *S. cerevisiae*.
(PDF)

Figure S7 **GO-term enrichment analysis for OrthoMCL clusters common to** ***D. bruxellensis, P. pastoris, P. angusta*** **, and** ***S. cerevisiae*** **.** Molecular function GO-terms enriched in comparison to *P. pastoris* and *P. angusta*, but not *S. cerevisiae*.
(PDF)

Figure S8 **GO-term enrichment analysis for OrthoMCL clusters common to** ***D. bruxellensis, P. pastoris, P. angusta*** **, and** ***S. cerevisiae*** **.** Biological process GO-terms enriched in comparison to *S. cerevisiae*, but not *P. pastoris* and *P. angusta*.
(PDF)

Figure S9 **GO-term enrichment analysis for OrthoMCL clusters common to** ***D. bruxellensis, P. pastoris, P. angusta*** **, and** ***S. cerevisiae*** **.** Cellular component GO-terms enriched in comparison to *S. cerevisiae*, but not *P. pastoris* and *P. angusta*.
(PDF)

Figure S10 **GO-term enrichment analysis for OrthoMCL clusters common to** ***D. bruxellensis, P. pastoris, P. angusta*** **, and** ***S. cerevisiae*** **.** Molecular function GO-terms enriched in comparison to *S. cerevisiae*, but not *P. pastoris* and *P. angusta*.
(PDF)

Table S1 Contig statistics. Average sequence coverage and length of contigs ordered by position within scaffolds.
(XLS)

Table S2 *D. bruxellensis* open reading frame ortholog group assignments. OrthoMCL assignments and sequence ID of best hit for all predicted protein coding open reading frames.
(XLS)

Table S3 Putative orthologs of *D. bruxellensis* proteins across 20 fungal species. The proteomes of species frequently assigned as top blastp hit for *D. bruxellensis* proteins in Blast2Go were used in pairwise blastp against the *D. bruxellensis* proteome, and reciprocal top-hits identified as putative orthologs.
(XLS)
