# Supplementary figures and images for: De-Novo Assembly and Analysis of the Heterozygous Triploid Genome of the Wine Spoilage Yeast Dekkera bruxellensis AWRI1499
Source: PLoS One. 2012 Mar 28;7(3):e33840. doi: 10.1371/journal.pone.0033840 (PMC3314683; doi:10.1371/journal.pone.0033840)

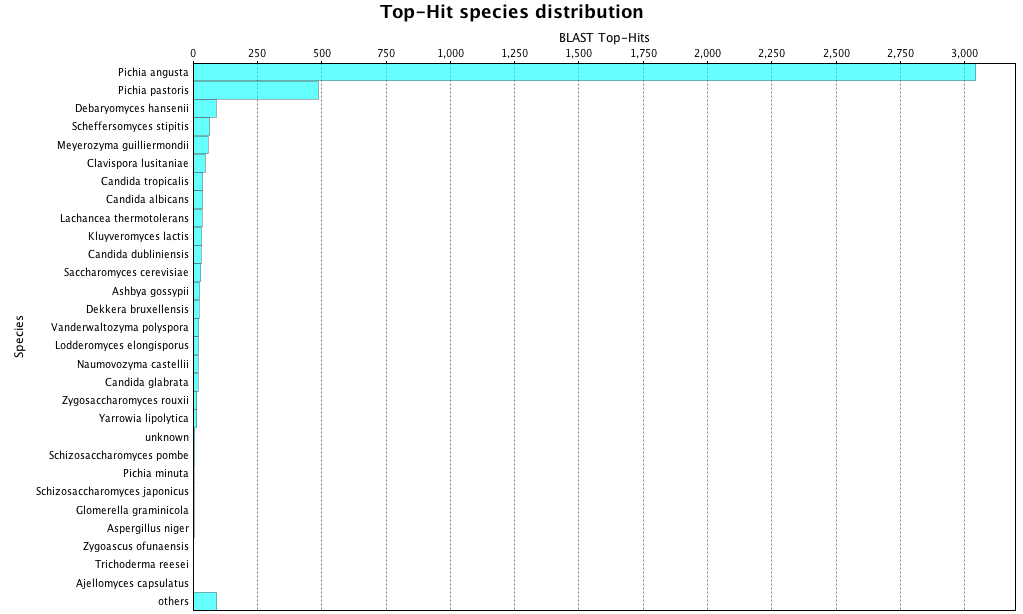

Supplement: Figure S1 — Distribution of Blastp top-hits against non-redundant database performed by Blast2go for all predicted D. bruxellensis AWRI1499 proteins. (TIF) [file pone.0033840.s001.tif]

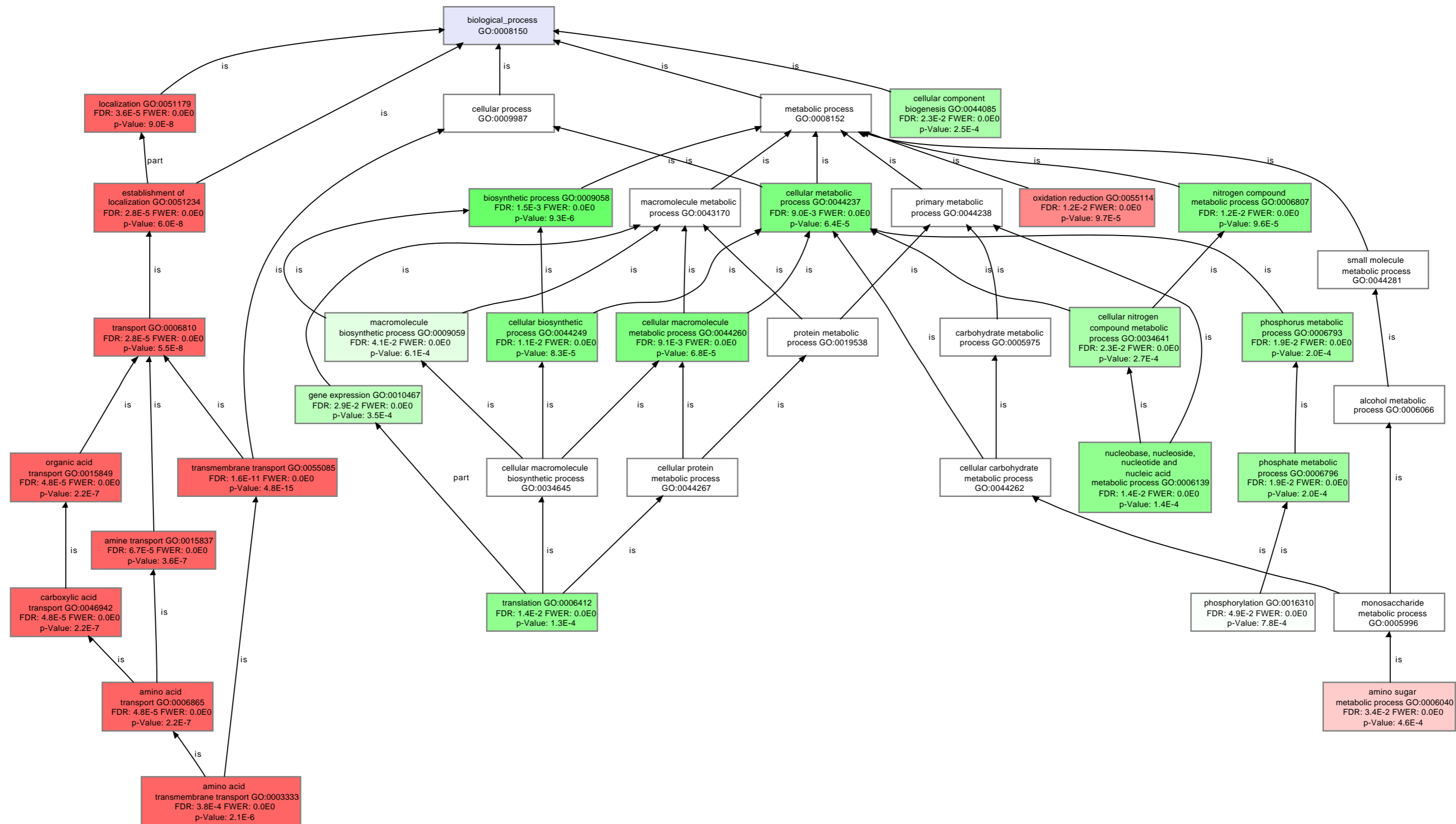

Enriched Graph

Supplement: Figure S2 — GO-term enrichment analysis for OrthoMCL clusters common to D. bruxellensis, P. pastoris, P. angusta, and S. cerevisiae . Biological process GO-terms enriched in comparison to all three spp. (PDF) [file pone.0033840.s002.pdf]

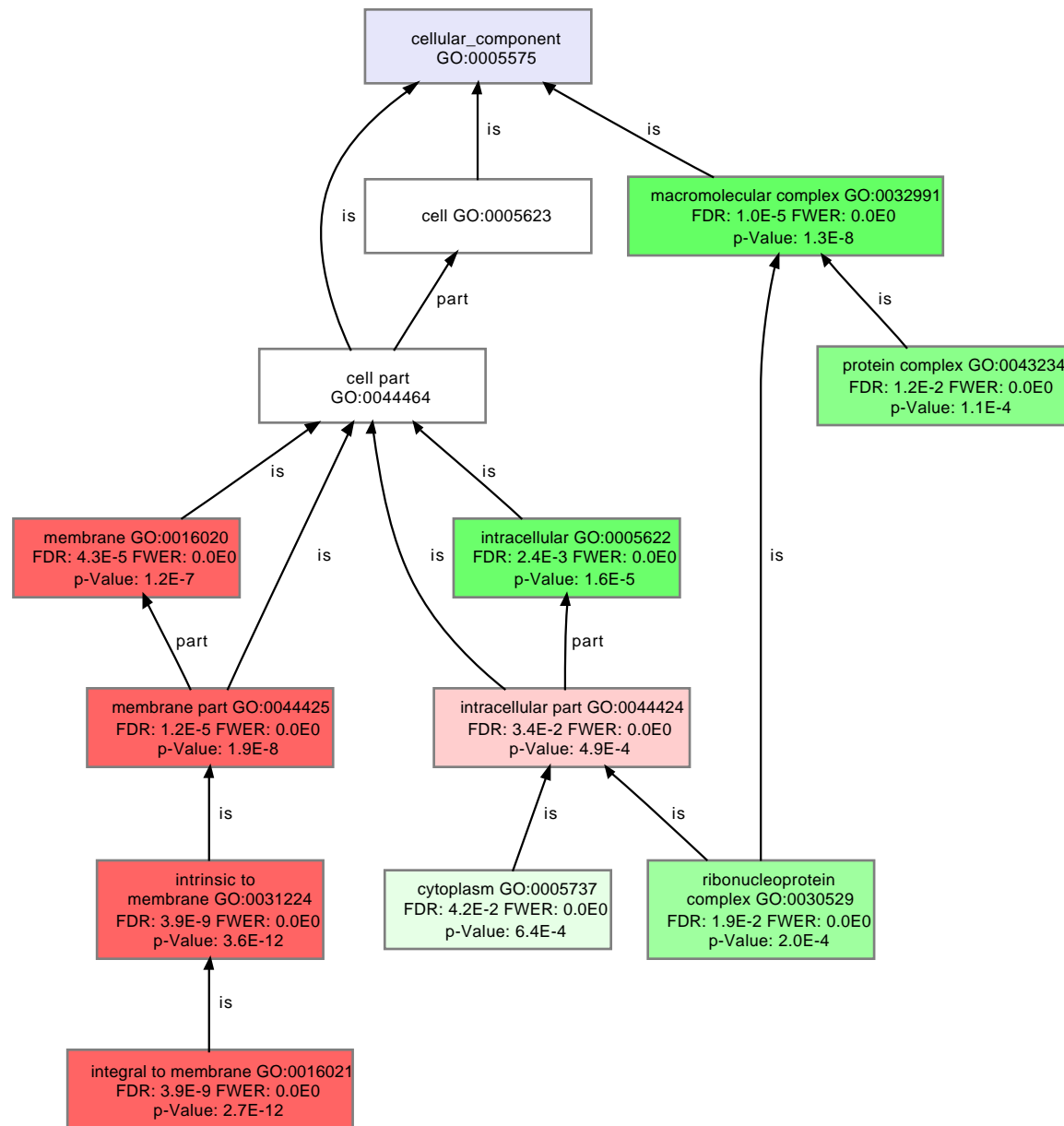

Enriched Graph

Supplement: Figure S3 — GO-term enrichment analysis for OrthoMCL clusters common to D. bruxellensis, P. pastoris, P. angusta , and S. cerevisiae . Cell component GO-terms enriched in comparison to all three spp. (PDF) [file pone.0033840.s003.pdf]

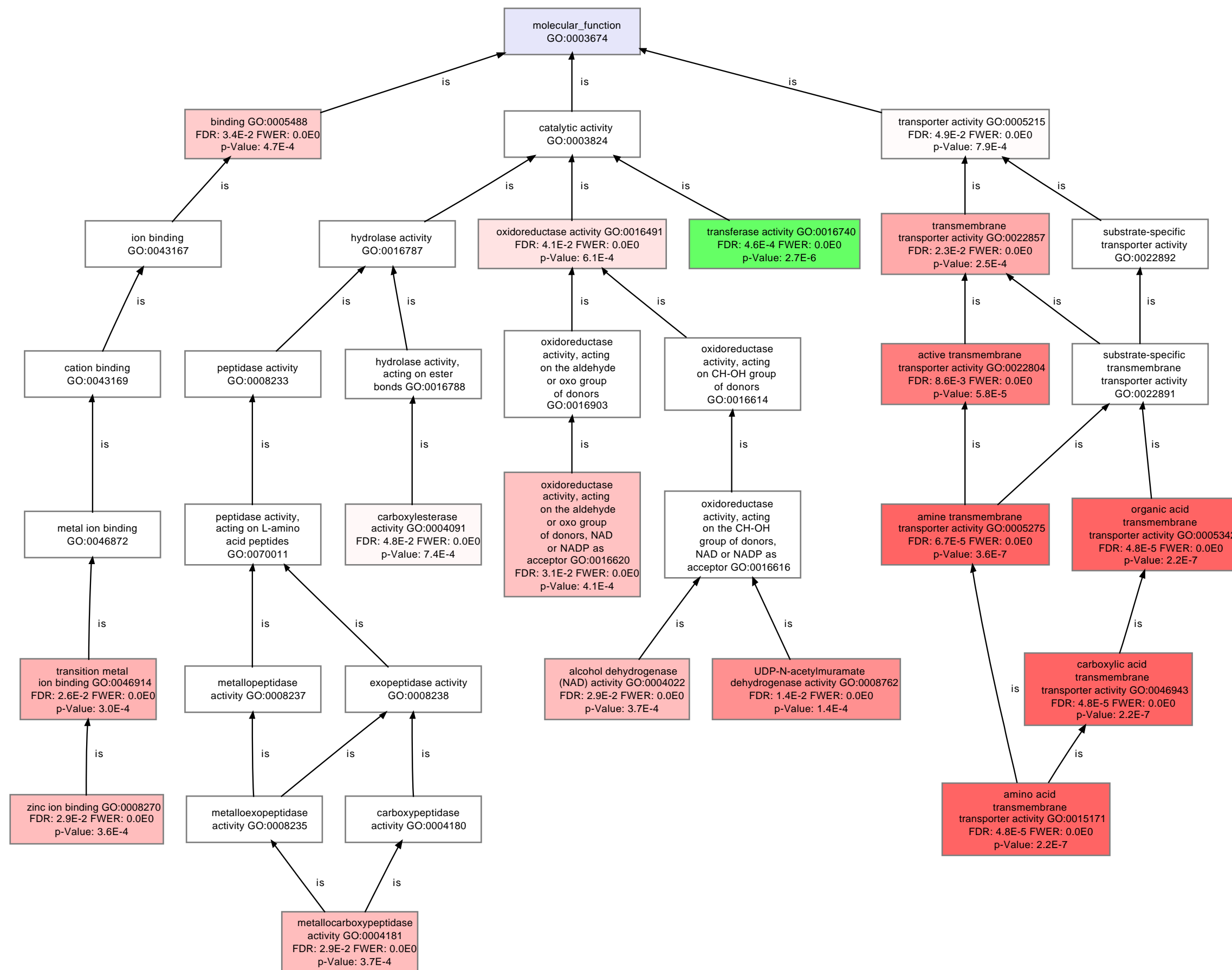

Enriched Graph

Supplement: Figure S4 — GO-term enrichment analysis for OrthoMCL clusters common to D. bruxellensis, P. pastoris, P. angusta , and S. cerevisiae . Molecular function GO-terms enriched in comparison to all three spp. (PDF) [file pone.0033840.s004.pdf]

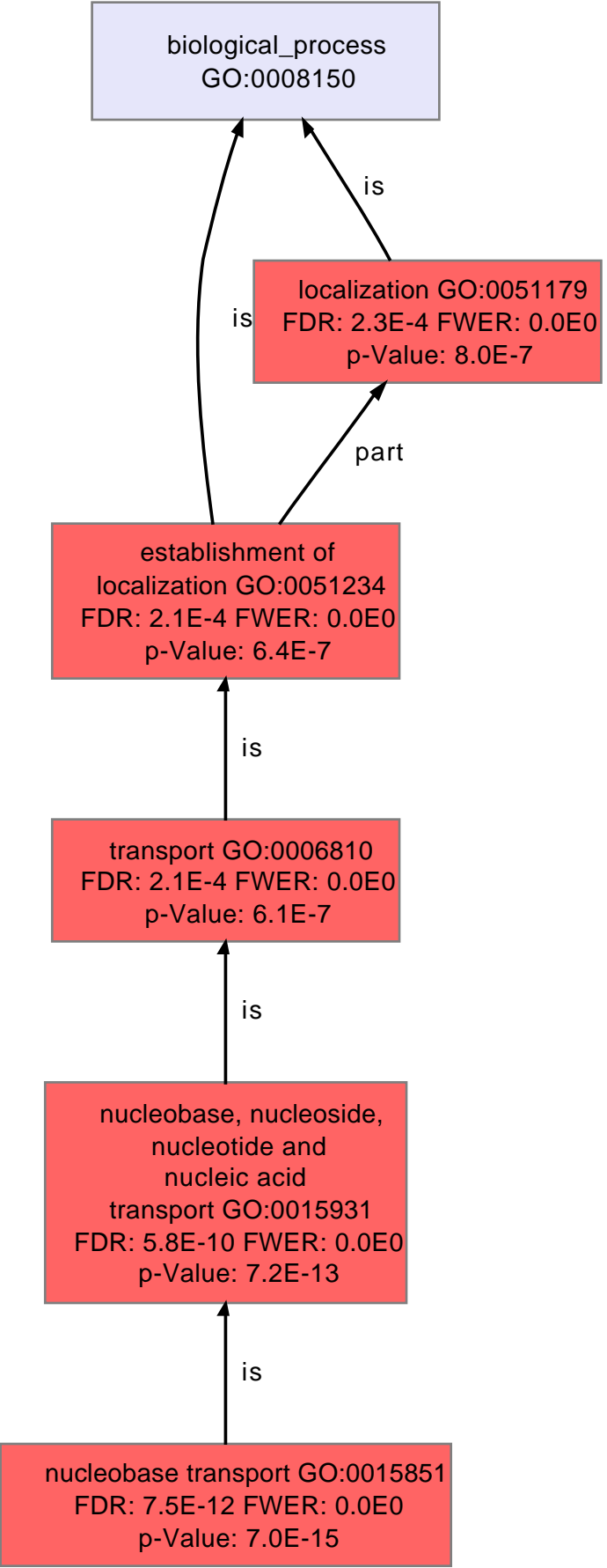

Enriched Graph

Supplement: Figure S5 — GO-term enrichment analysis for OrthoMCL clusters common to D. bruxellensis, P. pastoris, P. angusta , and S. cerevisiae . Biological process GO-terms enriched in comparison to P. pastoris and P. angusta, but not S. cerevisiae. (PDF) [file pone.0033840.s005.pdf]

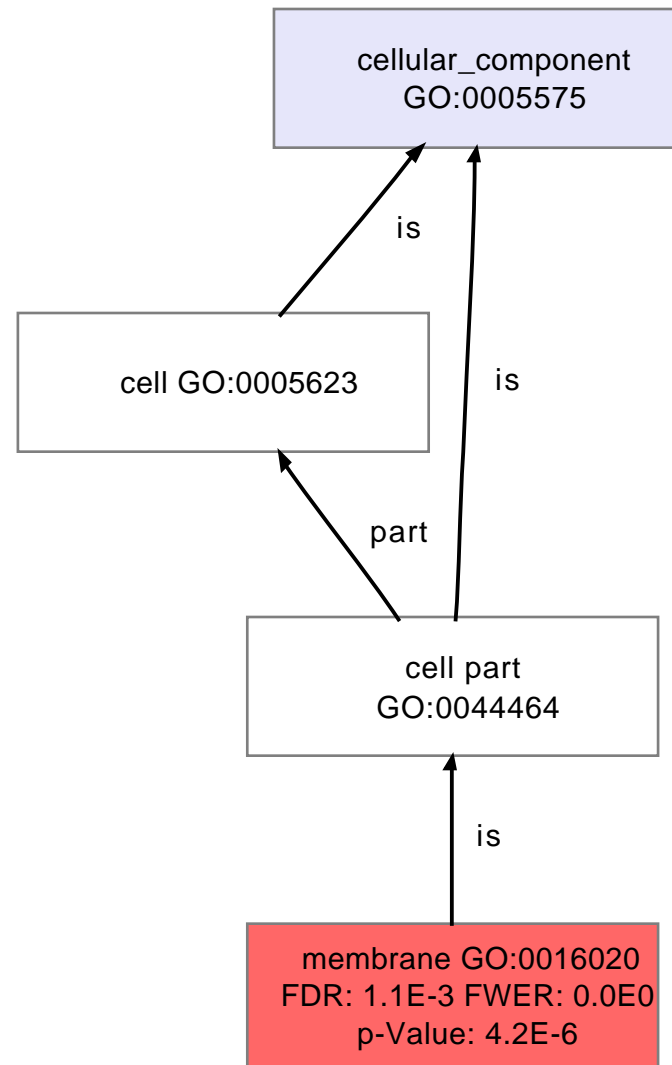

Enriched Graph

Supplement: Figure S6 — GO-term enrichment analysis for OrthoMCL clusters common to D. bruxellensis, P. pastoris, P. angusta , and S. cerevisiae . Cellular component GO-terms enriched in comparison to P. pastoris and P. angusta, but not S. cerevisiae. (PDF) [file pone.0033840.s006.pdf]

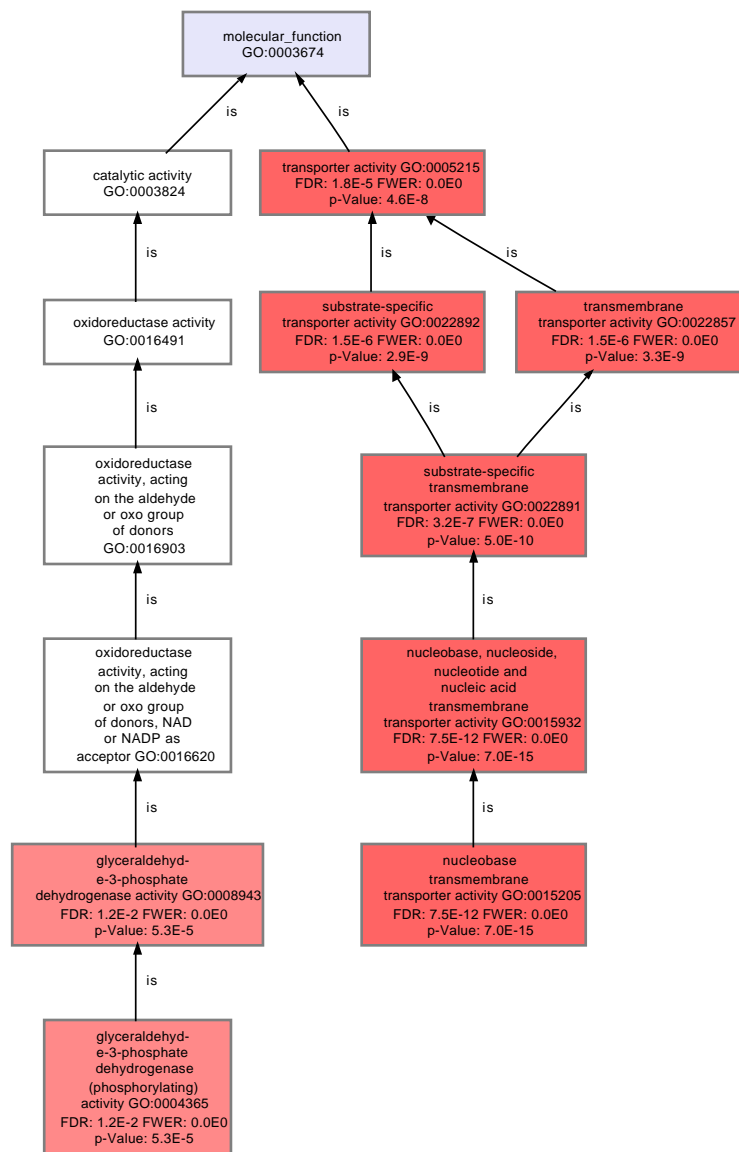

Enriched Graph

Supplement: Figure S7 — GO-term enrichment analysis for OrthoMCL clusters common to D. bruxellensis, P. pastoris, P. angusta , and S. cerevisiae . Molecular function GO-terms enriched in comparison to P. pastoris and P. angusta, but not S. cerevisiae. (PDF) [file pone.0033840.s007.pdf]

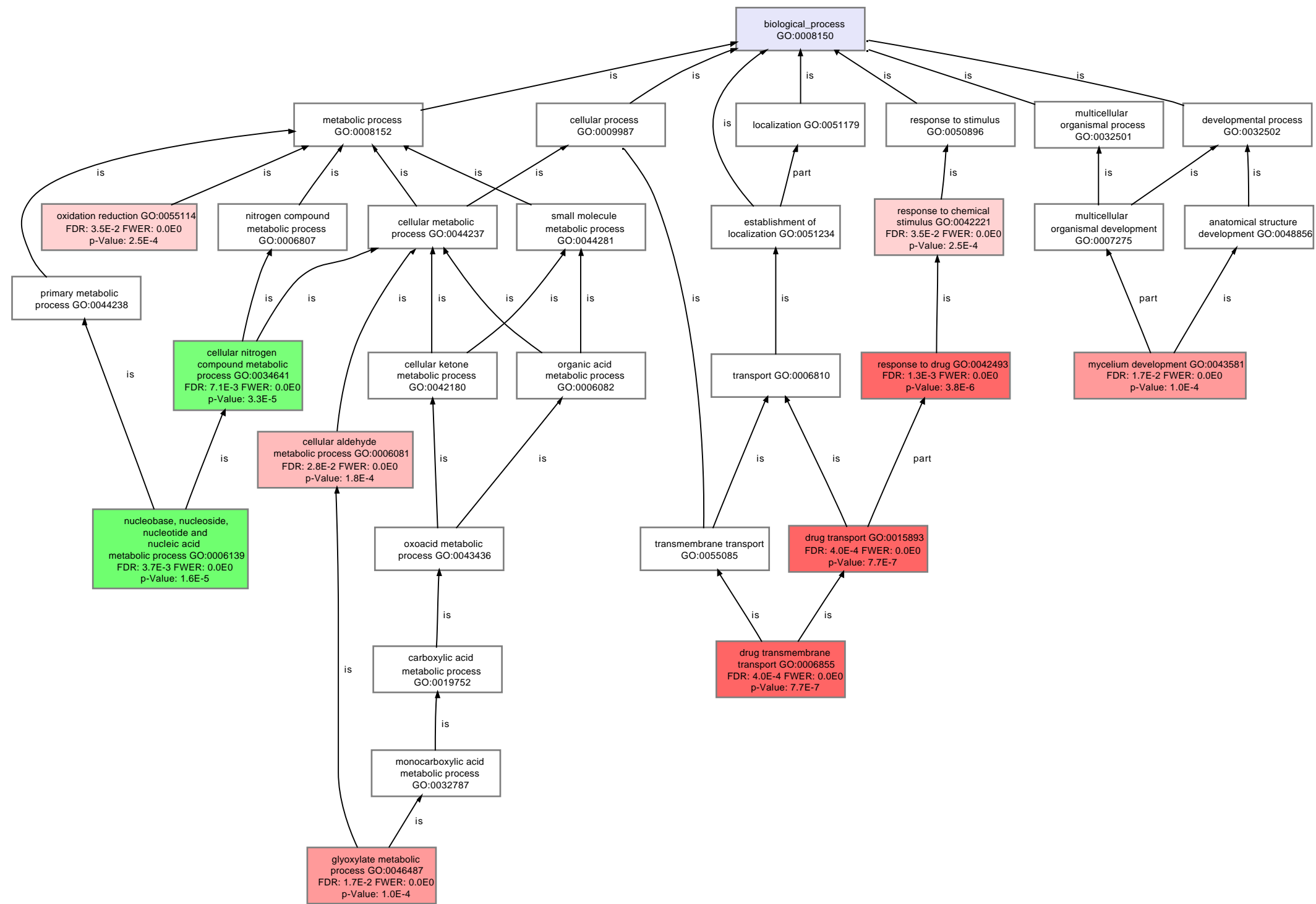

Enriched Graph

Supplement: Figure S8 — GO-term enrichment analysis for OrthoMCL clusters common to D. bruxellensis, P. pastoris, P. angusta , and S. cerevisiae . Biological process GO-terms enriched in comparison to S. cerevisiae, but not P. pastoris and P. angusta. (PDF) [file pone.0033840.s008.pdf]

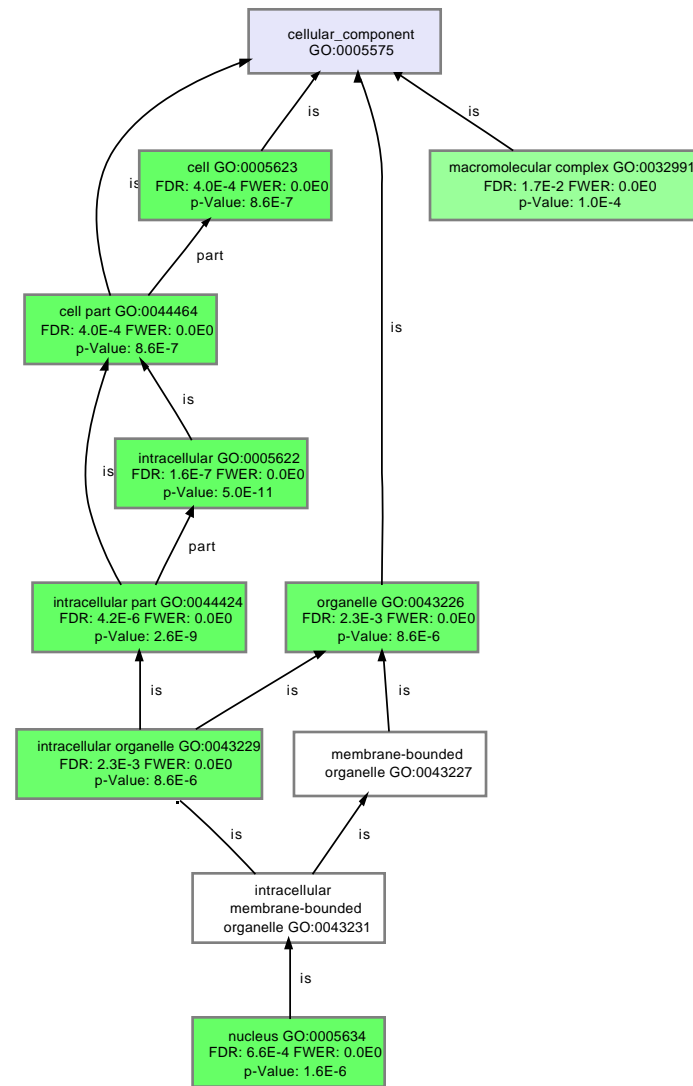

Enriched Graph

Supplement: Figure S9 — GO-term enrichment analysis for OrthoMCL clusters common to D. bruxellensis, P. pastoris, P. angusta , and S. cerevisiae . Cellular component GO-terms enriched in comparison to S. cerevisiae, but not P. pastoris and P. angusta. (PDF) [file pone.0033840.s009.pdf]

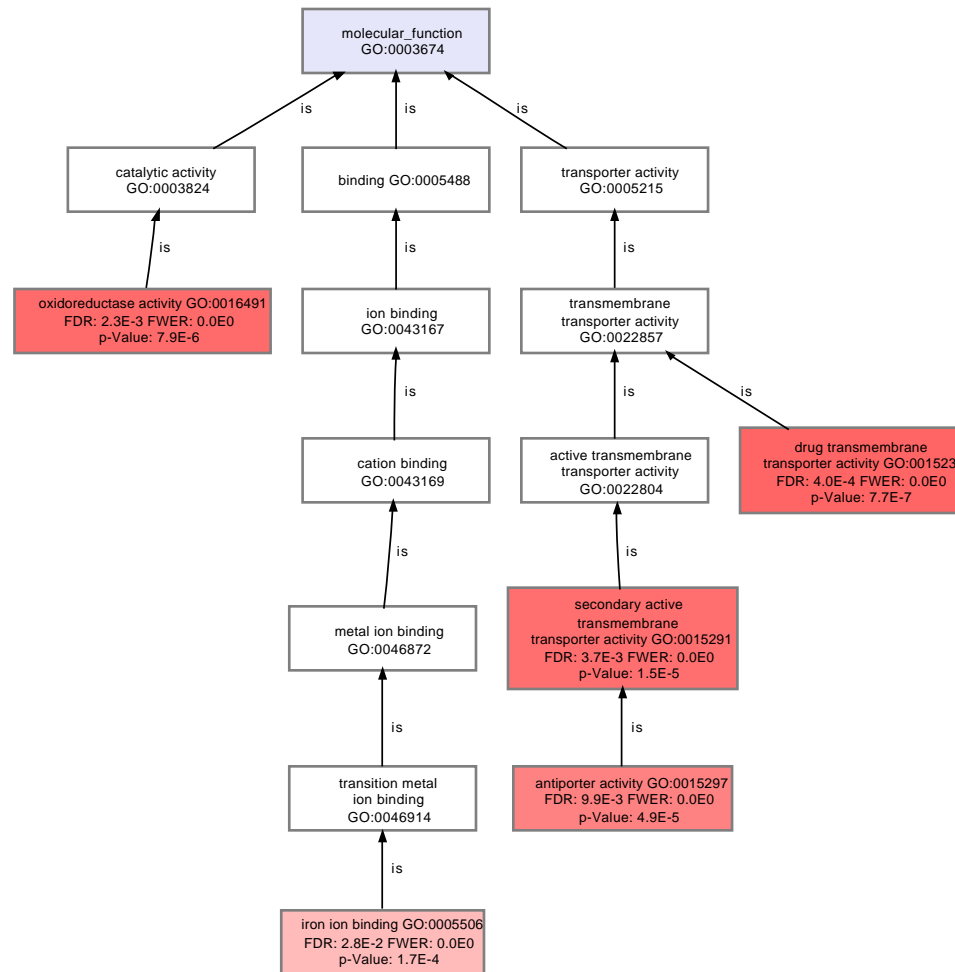

Enriched Graph

Supplement: Figure S10 — GO-term enrichment analysis for OrthoMCL clusters common to D. bruxellensis, P. pastoris, P. angusta , and S. cerevisiae . Molecular function GO-terms enriched in comparison to S. cerevisiae, but not P. pastoris and P. angusta. (PDF) [file pone.0033840.s010.pdf]
